# Outpatient total knee arthroplasty leads to a higher number of complications: a meta-analysis

**DOI:** 10.1186/s13018-020-01925-x

**Published:** 2020-09-14

**Authors:** Vittorio Bordoni, Alberto Poggi, Stefano Zaffagnini, Davide Previtali, Giuseppe Filardo, Christian Candrian

**Affiliations:** 1 Unità Ortopedica e Traumatologica, Ospedale Regionale di Lugano, EOC, Lugano, Switzerland; 2 II Clinica Ortopedica e Traumatologica, IRCCS Istituto Ortopedico Rizzoli, Bologna, Italy; 3 Applied and Translational Research Center, IRCCS Istituto Ortopedico Rizzoli, Bologna, Italy

**Keywords:** Total knee arthroplasty, TKA, Outpatient, Complications

## Abstract

**Background:**

Careful pre- and post-operative management can allow surgeons to perform outpatient TKA, making this a more affordable procedure. The aim of the present meta-analysis is to compare outpatient and inpatient TKA.

**Methods:**

A systematic search of the literature was performed in July 2020 on PubMed, Web of Science, Cochrane library, and on the grey literature databases. The papers collected were used for a meta-analysis comparing outpatient and inpatient TKA in terms of complication and readmission rates. Risk of bias and quality of evidence were defined according to Cochrane guidelines.

**Results:**

The literature search resulted in 4107 articles; of these, 8 articles were used for the meta-analysis. A total of 212,632 patients were included, 6607 of whom were TKA outpatients. The overall complication rate for outpatient TKAs was 16.1%, while inpatient TKAs had an overall lower complication rate of 10.5% (*p* = 0.003). The readmission rate was 4.9% in outpatient TKAs and 5.9% in inpatient TKAs. Only 3 studies reported the number of deaths, which accounted for 0%. The included studies presented a moderate risk of bias, and according to GRADE guidelines, the level of evidence for complications and readmissions was very low.

**Conclusions:**

This meta-analysis documented that outpatient TKA led to an increased number of complications although there were no differences in the number of readmissions. However, future high-level studies are needed to confirm results and indications for the outpatient approach, since the studies currently available have a moderate risk of bias and a very low quality of evidence.

## Background

Over the past few years total knee arthroplasty (TKA) procedures benefited from an improvement of both surgical and anesthetic techniques, as well as perioperative care [1, 2], making it possible not only to improve clinical results, but also to reduce hospitalization length [3]. The number of surgical procedures increased in the past few years, with 600,000 procedures per year only in the USA, and it is expected to further grow in the future [4–6]. Accordingly, an improvement in the management with a faster recovery and shorter hospitalization could benefit patient in terms of higher satisfaction and better clinical outcomes, as well as society in terms of economic saving [7–10]. Protocols for fast-track procedures requiring only 2–3 days of hospitalization have already been established [11]. However, following the trend of other surgical specialties [12–15], the increasing need for cost saving in the last years led to an even faster management, with patient discharged the same day of the intervention.

Outpatient procedures are wide spreading among several care centers [16, 17]. Careful pre- and post-operative management can allow surgeons to perform outpatient TKA, making this a more affordable procedure. In fact, the usefulness in terms of cost saving has been documented in several studies [9, 18]. Moreover, discharging patients on the same day of the intervention is well accepted by the patients, increasing patient satisfaction and producing a lower perception of disease [15, 19, 20]. On the other hand, a possible disadvantage of outpatient TKA is that the faster discharge may hinder the monitoring of the immediate post-operative phases, which in turn may lead to an increased rate of complications and readmissions compared to the traditional inpatient approach. Therefore, outpatient TKA procedures remain controversial, and not all knee surgeons choose them. In this light, the understanding of the real risks in terms of complications would help physicians to better ascertain advantages/disadvantages while considering the management of TKA patients with an outpatient procedure.

Aim of this meta-analysis was to quantitatively evaluate and compare complication and readmission rates in outpatient and inpatient TKAs. The study hypothesis is that outpatient TKA leads to a higher number of complications and readmissions.

## Materials and methods

### Literature search strategy

A systematic search of the literature was performed on the 6th of July 2020 on PubMed, Web of Science, Cochrane library, and on the grey literature databases (clinicaltrials.gov, greylit.org, isrctn.org, and opengrey.eu). The following string was used: outpatient OR same-day AND arthroplasty OR replacement OR prosthesis AND complication OR readmission. The lists of references of the included articles were also manually reviewed to find more articles.

### Inclusion and exclusion criteria in the selection process

Inclusion criteria were written in English and focused on the comparison between outpatient and inpatient TKAs in terms of complication and readmission rates. When articles referred to the same database, only the study with more patients was included. Two authors (V.B. and A.P.) independently selected the articles. Titles and abstracts were used for the first screening, and articles that were thought to be included in this study were then read by both authors. When the two authors disagreed on whether to include a study, consensus was reached by discussion and by consultation with a third reviewer (G.F.). Review articles, meta-analyses, case reports, surgical technique articles, editorials, letters to the editor, preclinical studies, and studies not available in English were excluded. The PRISMA guidelines (Preferred Reporting Items for Systematic Reviews and Meta-Analyses) were used to conduct the study selection process [21].

### Study quality assessment

The risk of bias was assessed in non-randomized studies using the non-randomized studies of interventions (ROBINS-I) tool approved by the Cochrane collaboration. Low, moderate, or high risk of bias were determined on the basis of confounding bias, selection bias, bias related to classification of interventions, bias related to deviations from intended interventions, bias related to missing data, bias in the measurement of the outcome, and bias in the selection of the reported results.

The overall quality of evidence for each outcome was graded according to the Grading of Recommendations Assessment, Development and Evaluation (GRADE) guidelines. Two reviewers (V.B. and A.P.) independently assessed the quality of the studies included. Discrepancies were discussed and, if necessary, resolved by a third author (G.F.). Quality of evidence was defined as high, moderate, low, or very low on the basis of risk of bias, inconsistency, indirectness, imprecision, and publication bias.

### Data extraction strategy

An electronic table for data extraction was created prior to the study. Information was extracted about the demographics of the included patients, such as age, sex, and BMI, and about the study design, such as inclusion and exclusion criteria, number of patients included, number of outpatient and inpatient TKAs, type of surgical approach performed, and follow-up duration. The outcomes considered for the meta-analysis were complication and readmission rates (in a period of up to 12 months).

### Data synthesis and presentation

The comparison between outpatient and inpatient procedures was assessed with the Mantel-Hanszel test and expressed as risk ratios for complication and readmission rates (RR = risk ratio early/delayed). Heterogeneity was tested using Cochran’s Q statistic and *I*^2^ metric and was considered significant for *I*^2^ > 25%. A fixed-effect model was preferred in the absence of significant heterogeneity; otherwise, a random-effect model was used. The level of significance was set at *p* < 0.05.

## Results

### Review statistics

**Fig. 1:**
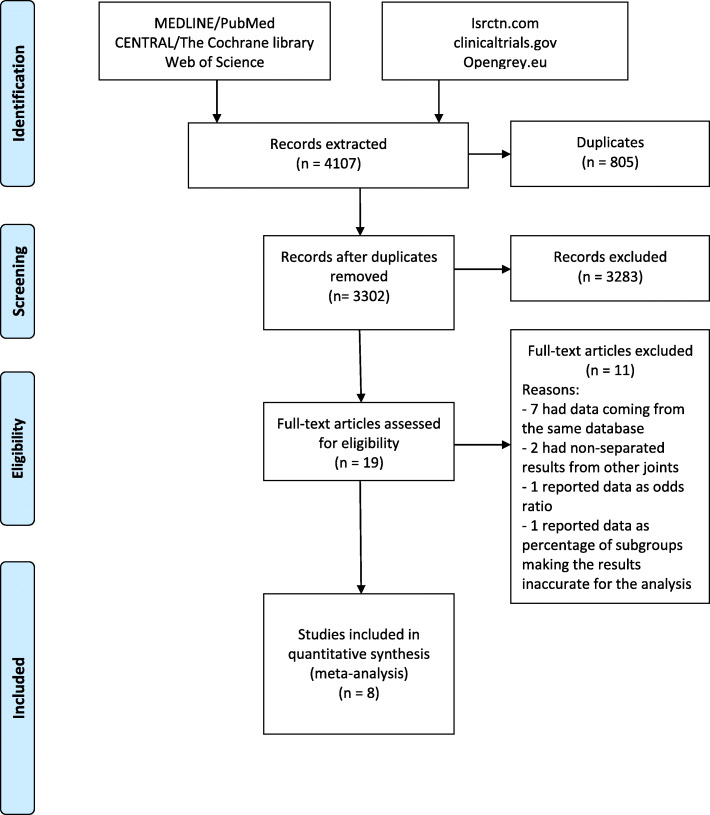
PRISMA flowchart. PRISMA (Preferred Reporting Items for Systematic Meta-Analyses) flowchart of the study selection process

The database search resulted in 4107 articles, 805 of which were duplicates. Of the remaining 3302 articles, 19 were suitable for inclusion. Eleven of these studies were excluded after full-text reading for the following reasons: 2 studies had results non-separated from other joints; 7 studies had data coming from the same database of other articles and included a lower number of patients; 1 study reported complications and readmission as odds ratio, 1 study reported data as percentage of subgroups making the results inaccurate for the analysis [22]. For this reasons, only 8 articles were included in the meta-analysis [23–30] (Fig. 1). A total of 212,632 patients were considered, (64% of the patients who underwent TKA were females) 6607 of whom were outpatients and 206,025 were inpatients. Age ranged from 62.5 to 74. Two articles reported the ASA score mean, which ranged from 1.6 to 2.2 in outpatient TKA, and between 2.1 and 2.6 in inpatient TKA. Only two articles reported the type of surgical approach: in one article a medial para-patellar approach was performed, in another article both medial-patellar and sub-vastus approaches were performed. Further details are reported in Table 1.

**Table 1:** Details of the studies

Study	Inclusion criteria	Number of patients	Demographics m/f (mean age) (BMI)	Number of complications-readmissions-deaths	Follow-up (months)
Arshi et al. [23] J Bone Joint Surg Am	Patients underwent TKA in PDPR database. Code CPT 27447	O: 4,391 I: 128,951 Total: 133,342	O: 1,560/2831 (na) (na) I: 46,805/82,146 (na) (na)	O: 833-na-na I: 18,049-na-na	12
Cassard et al. [24] Orthop Traumatol Surg Res	Consecutive patients undergone TKA in the institute	O: 61 I: 513 Total: 574	O: 38/23 (65.4) (na) I: 251/262 (70.5) (na)	O: 5-2-0 I: 37-25-0	1
Darrith et al. [25] J.Arthroplasty	No specific BMI or age cutoff, in general patients were physiologically young, without medical comorbidities that required an inpatient admission	O: 46 I: 46 Total: 92	O: na (na) (na) I: na (na) (na)	O: 5-0-0 I: 5-0-0	3
Gauthier-Kwan et al. [26] J.Arthroplasty	patients undergone primary TKA for end-stage osteoarthritis an ASA of 3 or less with a stable medical profile, and a BMI under 45 kg/m2	O:43 I:43 tot:86	O:29/14 (62.5)(28.6) I:22/21 (62.5)(30.4)	O:8-1-na I:6-1-na	3
Gillis et al. [27] Int Orthop	Consecutive patients undergone TKA in the institute	O: 125 I: 275 Total: 400	O: 58/64 (62.9) (33.5) I: 107/168 (66) (28.8)	O: 17-3-0 I: 36-12-0	3
Kimball et al. [30] Orthopedics	18 years or older listed as outpatient or inpatient in their database	O: 863 I: 863 Total: 1726	O: 373/490 (na) (na) I: 373/490 (na) (na)	O: na-44-na I: na-63-na	3
Nowak et al. [28] Bone Joint J	18 years or older who underwent TKA between 2005 and 2016 using the ACS NSQIP database	O: 986 I: 75,260 Total: 76,246	O: 328/658 (67.5) (32) I: 27,105/48,155 (67.1) (31.8)	O: 44-na-na I: 2331-na-na	1
Springer et al. [29] Orthop Clin North Am	Healthy patients with no active cardiopulmonary conditions, no history of sleep apnea, deep venous thrombosis, or pulmonary embolus, BMI) less than 40, good family support at home	O: 92 I: 74 Total: 166	O: na (na) (na) I: na (na) (na)	O: 15-12-na I: 7-6-na	1

### Study quality assessment

The included studies were all non-RCTs: 7 had a retrospective design and 1 had a prospective design. All the studies presented a moderate risk of bias: in the retrospective studies, this was due to the selection of the patients based on the characteristics observed after the start of intervention, and in the prospective study, it was due to the risk related to deviations from intended interventions.

### Quantitative synthesis

**Fig. 2:**
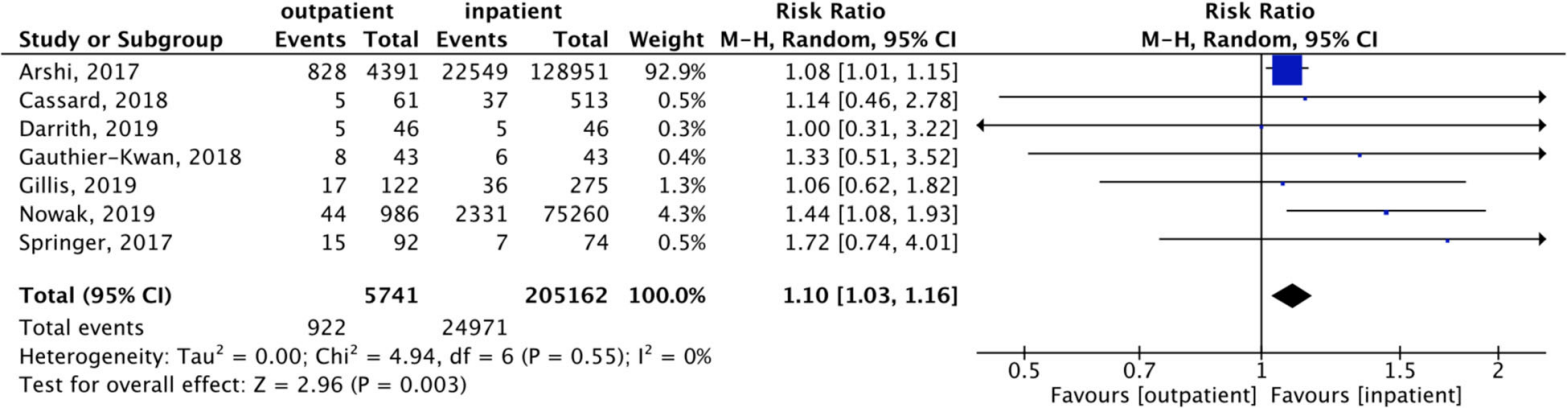
Complication rate. Forest plot of the complication rate comparing outpatient with inpatient TKA

Complications were reported in all the articles included, beside 1 study performed by Kimball et al. not reporting the overall complication rate [30]. The overall complication rate for TKA (both outpatient and inpatient) was 10.7%. The meta-analysis showed a statistically significant higher complication rate in outpatient TKA compared to inpatient TKA procedures (*p* = 0.003) (Fig. 2). More in detail, the comparative analysis of outpatient and inpatient TKA showed a complication rate of 16.1% for outpatient TKAs (from 4 to 19%), while a complication rate of 10.5% was documented in inpatient TKAs (from 3 to 17%). Only 2 studies distinguished complications as major or minor, thus plotting data for this sub-analysis was not possible. These studies showed that, among the reported complications, 49% (24/49 complications) were major in outpatient TKAs (2.3% in 1032 patients) and 33% (1153/2336 complications) were major in inpatient TKAs (1.5% in 75,306 patients), with the most frequently reported being heart attacks, infections requiring readmission, thrombosis, fractures, and mobilization of the prosthesis. Remaining complications were considered minor, with the most frequently reported being urinary infections, wound dehiscence, moderate anemia, and cutaneous rash.

**Fig. 3:**
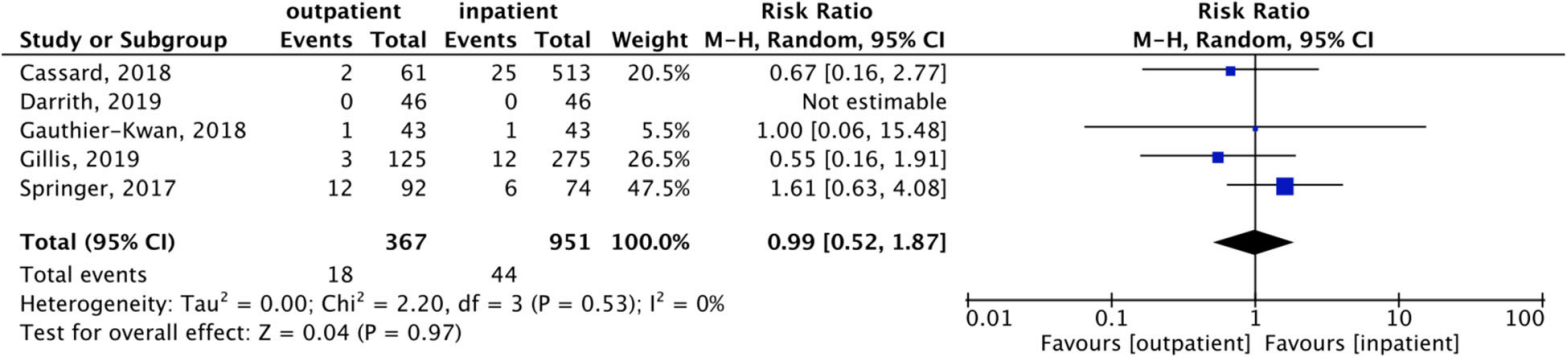
Readmission rate. Forest plot of the readmission rate comparing outpatient with inpatient TKA

The overall readmission rate was 5.6%. The meta-analysis showed a comparable readmission rate between the two approaches (Fig. 3). More in detail, outpatient TKAs had 4.9% readmissions, while inpatient TKAs had 5.9% readmissions. Only 3 studies reported the number of deaths, which was 0. Further details on complications and readmissions are reported in Table 1.

### Evidence of effectiveness

Accordingly, the level of evidence (LOE) for complications and readmissions resulted to be very low. In particular, the level of evidence of all the measured outcomes was low due to the non-randomized design, and it was further downgraded due to imprecision and indirectness.

## Discussion

The main finding of this meta-analysis is that outpatient TKAs lead to a slightly higher number of complications compared to inpatient TKAs. However, no difference was found in the readmission rate.

Outpatient TKAs are an emerging topic of interest at the center of the scientific discussion, as demonstrated by recent publications found by the literature search. In fact, all comparative studies are dated after 2017. The intense debate in this field is reflected by the diverging conclusions of the reported studies. Some authors suggested that no differences exist in terms of complication rate between outpatient TKAs and inpatient TKAs. The retrospective studies of Darrith et al. [25] on 92 patients (46 outpatients), Cassard et al. [24] on 574 patients (61 outpatients), Springer et al. [29] on 166 patients (92 outpatients), and Gillis et al. [27] on 400 patients (125 outpatients), as well as the prospective comparative cohort study of Gauthier-Kwan et al. [26] on 43 outpatients vs 43 inpatients TKAs, reported comparable complication and readmission rates. A comparable readmission rate was also reported by Kimball et al. [30] in a retrospective study analyzing 1726 patients (863 outpatients). On the contrary, other authors suggested that a faster discharge could lead to an increased number of complications: Nowak et al. [28] and Arshi et al. [23] evaluated respectively 76,246 and 133,342 patients (986 and 4391 outpatients, respectively), both reporting an increased number of complications among outpatient TKAs. Even though the available studies reach different conclusions, the overall results should be addressed critically. In fact, the studies supporting a similar number of complications are rather small retrospective series, likely underpowered, and thus methodologically not suited to give reliable findings. On the other hand, the studies underlying a higher number of complications focused their analysis on two large databases (American College of Surgeons National Surgical Quality Improvement Program and PearlDiver patient record database), which allowed to evaluate significantly bigger cohorts, with ten times more outpatient TKAs. Accordingly, the comparison of these large cohorts led to more reliable conclusions than the small retrospective series showing similar rates of complications, as confirmed by the different weights of the studies in the quantitative synthesis and by the conclusions of the meta-analysis.

This meta-analysis of the best available evidence documented an overall higher number of complications among patients who underwent outpatient procedures. While the overall results are of interest, it remains difficult to understand the relevance of these findings for the clinical practice, as the pooled risk ratio is only 1.1 and complications can range from simple cutaneous rashes to heart attacks. In this light, it would be paramount to distinguish major from minor complications. Unfortunately, only two studies reported their data with such details, which hindered the possibility to perform a sub-analysis, even though they suggested similar low rates of major complications. Beside the low number of studies, the study design might also introduce a bias in the reported findings. In fact, the retrospective design implies an uneven data collection, being based on medical charts that are more likely to report minor complications in inpatient TKAs rather than in outpatient TKAs. The outpatient procedure could not allow to identify complications that usually occur the first few days after the intervention. Therefore, the higher ratio of major complications observed in outpatient TKAs might be misleading, being likely affected by the presence of a selective reporting bias, with possibly underreported minor complications, rather than due to a real difference in the rate of events.

Readmission rate in this meta-analysis was found comparable between outpatient and inpatient TKAs in 6 out 8 studies reporting this data. This apparently divergent finding compared to the higher complication rate previously reported for outpatient TKA can be explained by different aspects. The two large databases did not report the readmission rate, leaving a smaller cohort of patients and therefore a likely underpowered analysis to address this issue. On the other hand, not all complications require a readmission, as they could be managed with other interventions. In this light, readmissions should not be considered as the only aspect of a financial planning, as the costs of managing complications should be weighted as well, being able to nullify the initial economic savings. To this regard, another important aspect that should be analyzed is that among the studies included in the present meta-analysis only two reported the destination of the patient after the discharge. The other articles did not report if patients were accepted by a nursing facility or if they went home. The nursing facility represents a high cost that must be added to the cost of the procedure and should be considered when an outpatient TKA is performed. All these aspects deserve further attention to properly address the question on the possible economic savings of outpatient TKA.

The meta-analysis on the current literature presents some limitations, mainly related to the low study level and to the heterogeneity of both studies and patients evaluated. Moreover, the selection bias in non-randomized comparative studies was found to be moderate, therefore the results of outpatient or inpatient TKA complications should be interpreted with caution. Similarly, the indication of discharge could have been related to different patient characteristics. This is a key point, since rather than defining if outpatient TKAs lead to more complications or readmissions, research efforts should be invested in the identifications of the most suitable candidates that can benefit without risks from the outpatient approach. Although some authors tried to define some essential characteristics for the patient in order to be managed as outpatient [31], there is still no consensus on the proper patient selection. Another weakness is the limited number of studies evaluated. Most of the studies in the literature reported on the same two large databases [23, 28] and therefore only the two larger studies could be included. Moreover, no RCTs were available, which affected the level of evidence. Nonetheless, despite the aforementioned limitations, the literature allowed to draw important conclusions.

This meta-analysis was able to investigate a large number of patients and demonstrated that the complication rate was slightly higher in outpatient TKAs compared to the inpatient approach, even though the readmission rate was similar. In this light, even if the magnitude of the difference between inpatient and outpatient TKA in terms of complication rate is limited, the documented higher number of complications among outpatient TKAs supports the need to better identify patients who can benefit from this procedure without risks. Future studies should consider both patient characteristics and a randomized design, in order to confirm advantages and disadvantages of outpatient TKA.

## Conclusion

This meta-analysis showed that outpatient TKA leads to a slightly increased number of complications, although there were no differences in the number of readmissions between the two procedures. The low-level of comparative studies, affected by a moderate risk of bias, and therefore the very low quality of evidence, underline the need of high-level studies to confirm these findings and identify the most suitable candidate for outpatient TKA.

## Data Availability

The datasets used and/or analyzed in the current study are available from the corresponding author on reasonable request.

## Abbreviations

TKA: Total knee arthroplasty
LOE: Level of evidence
